# Stoichiometry-Controlled Reversible Lithiation Capacity in Nanostructured Silicon Nitrides Enabled by *in Situ* Conversion Reaction

**DOI:** 10.1021/acsnano.1c06927

**Published:** 2021-09-27

**Authors:** Asbjørn Ulvestad, Marte O. Skare, Carl Erik Foss, Henrik Krogsæter, Jakob F. Reichstein, Thomas J. Preston, Jan Petter Mæhlen, Hanne F. Andersen, Alexey Y. Koposov

**Affiliations:** † Department of Battery Technology, Institute for Energy Technology, Instituttveien 18, NO-2027 Kjeller, Norway; ‡ Department of Materials Science and Engineering, Norwegian University of Science and Technology, Alfred Getz vei 2, NO-7491 Trondheim, Norway; § Center for Materials Science and Nanotechnology, Department of Chemistry, University of Oslo, P.O. Box 1033, Blindern, 0371 Oslo, Norway

**Keywords:** silicon-based materials, nanoparticles, pair distribution function, conversion anode, lithium-ion batteries

## Abstract

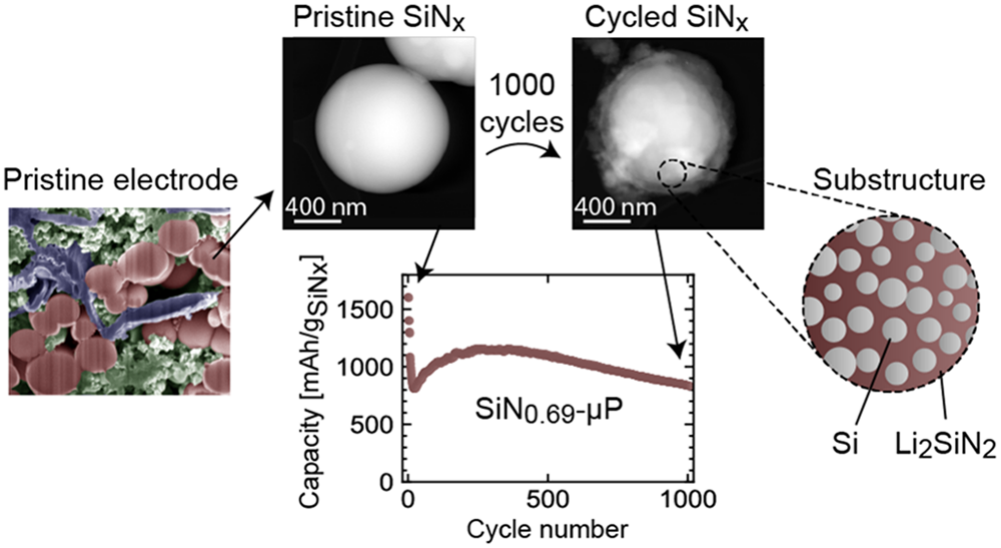

In modern Li-based batteries, alloying anode materials have the potential to drastically improve the volumetric and specific energy storage capacity. For the past decade silicon has been viewed as a “Holy Grail” among these materials; however, severe stability issues limit its potential. Herein, we present amorphous substoichiometric silicon nitride (SiN_*x*_) as a convertible anode material, which allows overcoming the stability challenges associated with common alloying materials. Such material can be synthesized in a form of nanoparticles with seamlessly tunable chemical composition and particle size and, therefore, be used for the preparation of anodes for Li-based batteries directly through conventional slurry processing. Such SiN_*x*_ materials were found to be capable of delivering high capacity that is controlled by the initial chemical composition of the nanoparticles. They exhibit an exceptional cycling stability, largely maintaining structural integrity of the nanoparticles and the complete electrodes, thus delivering stable electrochemical performance over the course of 1000 charge/discharge cycles. Such stability is achieved through the *in situ* conversion reaction, which was herein unambiguously confirmed by pair distribution function analysis of cycled SiN_*x*_ nanoparticles revealing that active silicon domains and a stabilizing Li_2_SiN_2_ phase are formed *in situ* during the initial lithiation.

## Introduction

The development of energy storage solutions capable of delivering high energy density and fast charging is a key element for the further implementation of electrification and decarbonization technologies. With this goal in mind, a significant effort has been devoted to developing electrode materials for the Li-ion batteries (LIBs) as LIBs are on the path of approaching the limit with a current materials set.^1^ With regards to anode materials, much of this effort has been focused on silicon (Si) as well as designing different pathways to overcome the cycling stability issues of Si that are currently preventing the widespread use of this otherwise promising anode material. The motivation for the extensive research in this field is primarily driven by the high theoretical lithium storage capacity of Si (3579 mAh g^–1^), compared with that of the currently used graphitic materials (372 mAh g^–1^), coupled with a low lithiation potential.^2^ The large specific capacity of Si, however, results in significant volumetric changes of Si during lithiation and delithiation, causing several failure mechanisms. These are related to fracturing^3−5^ and morphological changes^6−8^ of the Si material itself occurring during cycling and to instability of the solid electrolyte interphase (SEI)—the latter results in electrode densification and continuous lithium (Li) and electrolyte consumption, which are at limited supply in a battery cell.^9−12^

A typical method for preventing fracturing of Si and other alloying materials has generally been a dimensional stabilization, that is, reducing the size of the material below a critical fracturing threshold.^3^ Specialized combinations of dimensional stabilization coupled with engineered coatings have yielded materials with excellent performance, but this is generally accomplished though rather complex chemical processing.^13−16^ A different approach to overcome the stability issues of Si-based anodes is the use of conversion alloying materials. During the initial lithiation, these materials irreversibly convert into a mixture of an inactive matrix component and an active component. The reversible Li storage capacity in the subsequent cycles stems from the reversible lithiation and delithiation of such active component, which typically operates through alloying mechanism.^17−19^ Thus, the volumetric changes of an active component are buffered or confined by an inactive matrix mitigating the degradation pathways described above and ultimately leading to extended lifetime of the complete anodes. This group of materials was initially described in a patent application by Idota et al.,^20^ introducing the tin-based oxide composite electrode.^21^ The mechanism of its functionality was later elucidated by Courtney and Dahn using X-ray diffraction confirming the formation of metallic tin as an active component through initial lithation.^22^ The principle of conversion of materials into active and inactive components during the first cycle has since been extended to other materials such as silicon oxide (SiO_*x*_),^17,23−28^ the most well-known representative within the class of conversion alloying materials at the present moment. More recently, a similar approach was used for the design and subsequent preparation of amorphous and substoichiometric silicon nitride (SiN_*x*_) thin films.^18,19,29−33^ These preliminary investigations of SiN_*x*_ thin film electrodes have shown rather intriguing combinations of relatively high gravimetric capacities and potentially promising cycling stability. However, with one notable exception,^34^ validation of SiN_*x*_ performance as a materials in more practical particle-based composite electrodes, which could be prepared through convention slurry-based process, as well as general understanding of their functionality is lacking.

Such gap in the synthesis, fundamental understanding, application, and evaluation of SiN_*x*_ materials under more practical circumstances is remedied in the present paper, where we demonstrate the performance and characteristics of anodes for LIBs based on amorphous and substoichiometric silicon nitride (SiN_*x*_) nanoparticles of different chemical compositions and particle’ sizes. The studied materials and respective electrodes were thoroughly characterized in pristine conditions and at different stages of cycling. Furthermore, synchrotron pair distribution function (PDF) analysis was conducted *ex situ* to elucidate the structural changes taking place in SiN_*x*_ material during the conversion reaction through the initial cycle.

## Results and Discussion

### Synthesis of SiN_*x*_ and Structural Considerations

**Figure 1:**
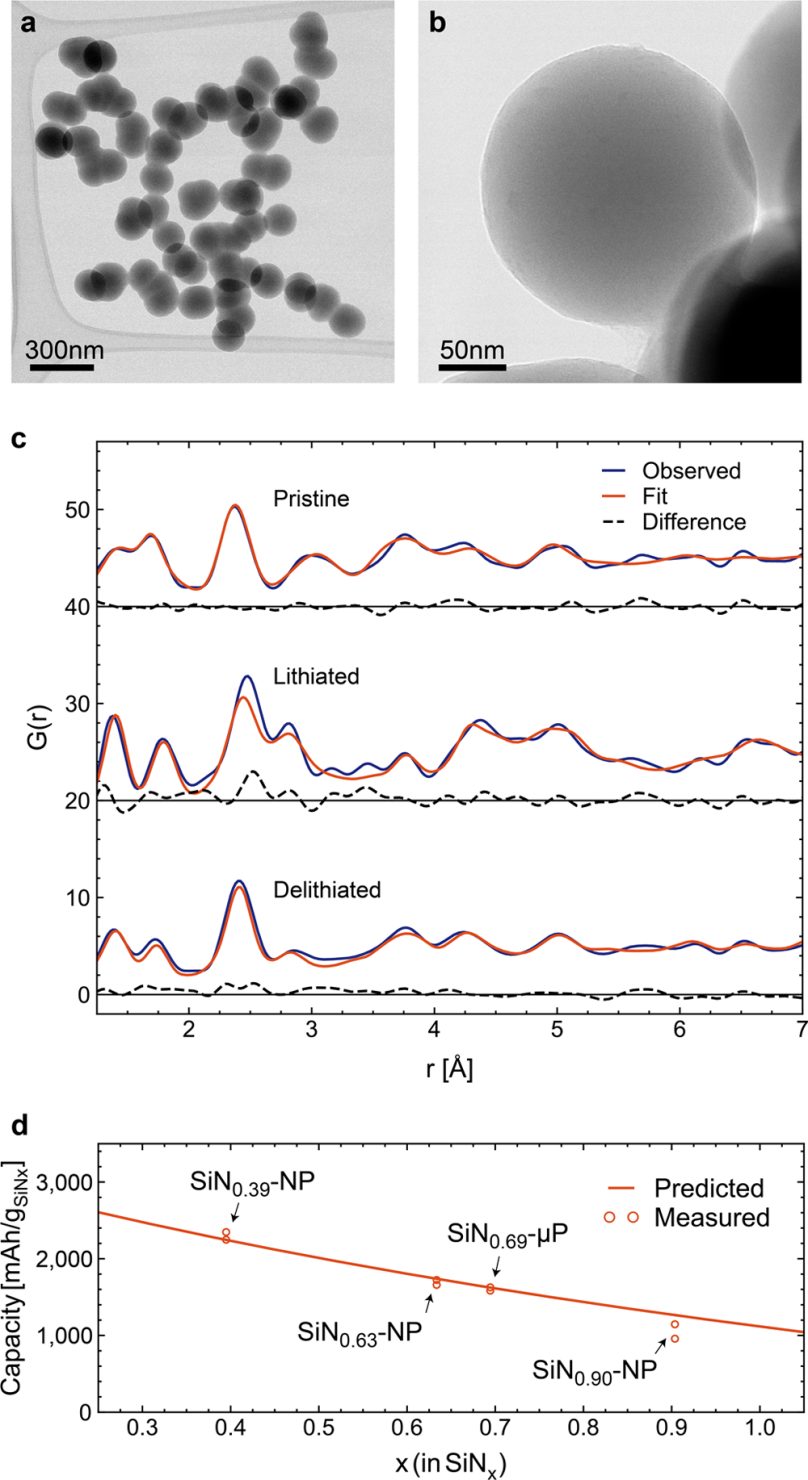
Characterization of particles and first cycle conversion characteristics: (a, b) bright-field STEM images of SiN_0.63_-NPs; (c) PDF data from the electrode material containing 60 wt % SiN_0.63_-NP before cycling, after lithiation and after delithiation shown together with the best fit; (d) the reversible capacity in the first cycle of SiN_*x*_ of different compositions compared to the calculated capacity based on the determined conversion reaction (the shown measured capacities were obtained from two parallel cells fabricated for each chemical composition).

Amorphous SiN_*x*_ nanoparticles of several sizes and stoichiometries were synthesized by copyrolysis of ammonia (NH_3_) and silane (SiH_4_) through homogeneous nucleation in the gas phase using a free space reactor.^35^ Such a method has been successfully demonstrated for the preparation of nanoparticles of pure Si and allows to efficiently control not only the sizes of nanoparticles but also their morphologies by controlling nucleation and growth in the gas phase.^36^ For the synthesis of SiN_*x*_, the reaction temperature was selected to target the preparation of amorphous particles, and precursor’s ratio and flow rate were adjusted to synthesize particles of different sizes and chemical compositions. Within the present work, four examples of SiN_*x*_ were selected for further study to elucidate the influence of nitrogen content (chemical composition) and particle size on the electrochemical performance and stability of the prepared materials. The following sample nomenclature is used in the present article: SiN_*x*_-NP/μP, where *x* reflects the stoichiometry of the particles, NP or μP reflects the average size of the particles–nanoparticles or microparticles, respectively. Three selected samples had similar primary particle sizes and gradually increasing nitrogen content, as determined by TEM energy dispersive spectroscopy (EDS), SiN_0.39_-NP (D_v50_ = 184 nm), SiN_0.63_-NP (D_v50_ = 182 nm), SiN_0.90_-NP (D_v50_ = 184 nm)—to evaluate the effect of stoichiometry. One additional sample was selected for evaluation of the size effects and had a significantly larger particle size and an intermediate nitrogen content, SiN_0.69_-μP (D_v50_ = 952 nm). The prepared materials consisted of partially agglomerated spherical primary nanoparticles, as exemplified by the scanning transmission electron microscopy (STEM) images in Figure 1a,b for the sample SiN_0.63_-NP. Complementary scanning electron microscopy (SEM) images are shown in Figure S1. TEM-EDS mapping of all pristine SiN_*x*_ materials demonstrated that the particles contain a thin native oxide layer because of air exposure after the preparation (Figure S2). In addition, X-ray diffraction (XRD) analysis confirmed the expected amorphous structure of the materials (Figure S3).

The earlier investigations of SiN_*x*_ materials, using thin films as a model, have proposed that the initial lithiation of SiN_*x*_ results in the irreversible formation of an inactive matrix with a likely composition of Li_2_SiN_2_ along with clusters of lithiated Si, which can be subsequently delithiated and further participate in the electrochemical activity of the electrode.^18,19^ To verify this hypothesis and gain additional insight into this irreversible *in situ* conversion reaction, we conducted synchrotron X-ray pair distribution function (PDF) analysis of SiN_0.63_-NP. We believe that is reasonable to assume that the principles of the conversion mechanism/reaction will not depend on stoichiometry or the size of the particle. Therefore, for assessment of the conversion mechanism, the sample of SiN_0.63_-NP was studied in the pristine state (in a form of nanoparticles), as a baseline, and then in actual electrodes–prior cycling, after the first lithiation and after the subsequent delithiation (Figure S4). For the study of SiN_0.63_-NPs in the electrodes, the electrodes were prepared (with 60 wt % of SiN_0.63_-NP), and then the active layer was removed from the surface of the copper current collector in the inert atmosphere at different stages of cycling and encapsulated in the capillaries for PDF analysis (further details in SI). While fitting of amorphous phases requires techniques that are beyond the scope of this study, a fitting of the short-range (below 7 Å) correlations of the crystalline analogues of the potentially expected phases was conducted (Figure 1c). The fitting of the results was conducted using the reported crystalline phases of the expected compounds, that is, c-Si, α-Si_3_N_4_, and graphite for the uncycled material; Li_13_Si_4_, Li_7_Si_3_, Li_2_SiN_2_, LiC_6_, and LiC_12_ for the lithiated material; Si, Li_2_SiN_2_, graphite, and LiC_12_ (in case of residual lithiation) for the delithiated material. Based on the best fitting, PDF analysis unambiguously revealed that Li_2_SiN_2_ matrix is formed during initial lithiation and remains after subsequent delithiation, as was previously hypothesized.^18^ The consideration of other possible nitrogen-containing matrix phases in the analysis (Li_3_N, LiSi_2_N_3_ and Si_3_N_4_) did not provide a good fit of the experimental data. Furthermore, the PDF pattern of the lithiated SiN_0.63_-NP did not contain the characteristic features of fully lithiated silicon (Li_15_Si_4_), as was evidenced by the absence of the characteristic peak at 4.75 Å,^37^ but was dominated by the intermediate phases Li_7_Si_3_ and Li_13_Si_4_ instead. This demonstrates that the active Si domains in SiN_*x*_ are effectively self-limiting in lithiation despite cycling conditions similarly to silicon suboxides (SiO_*x*_).^27^

The knowledge obtained about the *in situ* conversion reaction (with a confirmed formation of Li_2_SiN_2_ as inactive matrix) allows predicting the delithiation capacities of the SiN_*x*_ materials based on their stoichiometry. Therefore, considering the compositional information for the pristine materials obtained from TEM-EDS, the samples SiN_0.90_-NP, SiN_0.63_-NP, SiN_0.39_-NP, and SiN_0.69_-μP were predicted to have delithiation capacities of 1265, 1738, 2245, and 1623 mAh g^–1^, respectively (the details of calculations are shown in SI). These predictions well correlate with the corresponding first cycle delithiation capacities, which were measured experimentally, as illustrated in Figure 1d. A plausible explanation for the slight overestimation of the delithiation capacity of SiN_0.90_-NP is the higher fraction of the inactive Li_2_SiN_2_ phase compared with the other samples with lower nitrogen content. That results in a higher conversion resistance in accordance with the previous observations.^29,32^

Prediction of capacities should also allow the estimation of anticipated Coulombic efficiencies (CEs) for the first cycle. However, a direct comparison of the predicted and measured lithiation capacity and CE is convoluted by surface contributions to the irreversible capacity as the conversion reaction mechanism allows only the prediction of the bulk properties of the material. This is reflected in lower-than-anticipated first cycle CEs, which are measured to be 46%, 57%, 66%, and 56% for samples SiN_0.90_-NP, SiN_0.63_-NP, SiN_0.39_-NP, and SiN_0.69_-μP, respectively, compared with corresponding CE values of 68%, 79%, 88%, and 77% predicted from the conversion reaction. The values of CE lower than could be estimated from the conversion process are mainly due to formation SEI, resulting in irreversible consumption of Li. It is expected though, that the first cycle CE can be improved to nearly predicted values with a relatively minor effort, for example, by adding a carbon coating, as reported for similar materials by Chae et al.^34^ Within the present work, no additional modifications of the materials have been conducted to minimize the number of influencing parameters and evaluate the chemistry of these materials in a pristine state.

### Electrochemical Stability of SiN_*x*_

**Figure 2:**
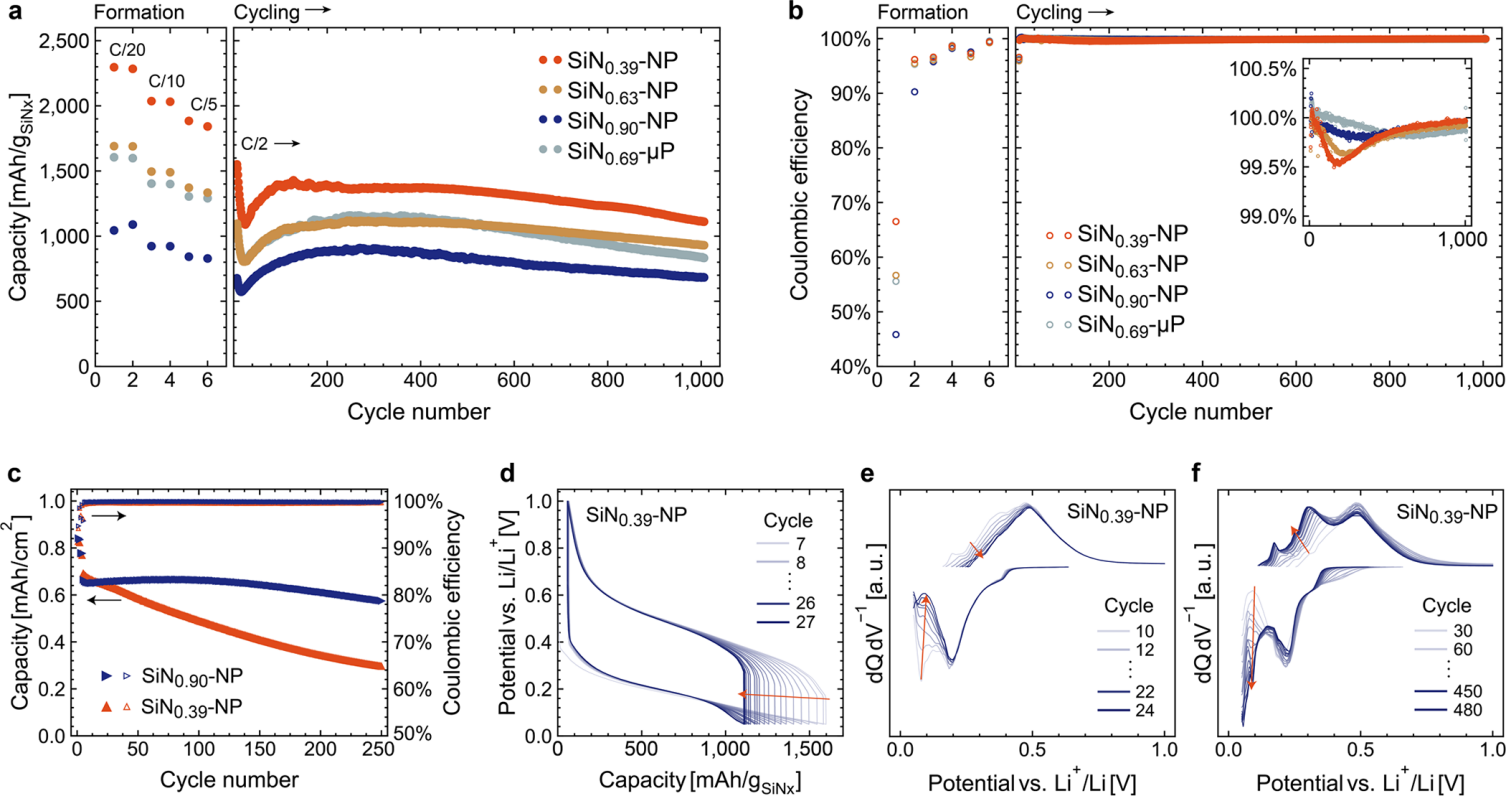
Electrochemical cycling of electrodes containing 60 wt % amorphous SiN_*x*_ particles of different composition and particle size: (a) cycling stability and (b) Coulombic efficiency (CE) of half cells over 1000 cycles with the inset showing the CE variations close to 100%; (c) capacity and CE of SiN_0.90_-NP and SiN_0.39_-NP in full cells against LFP cathodes cycles at C/2; (d) voltage *vs* capacity plot for SiN_0.39_-NP containing electrodes; (e) differential capacity analysis of SiN_0.39_-NP containing electrodes during the initial capacity drop; and (f) the differential capacity analyses during the capacity recovery in the subsequent cycles for SiN_0.39_-NP containing electrodes.

The obtained materials were further subjected to evaluation of their long-term cycling stability which was initially performed in half cells using Li as a counter electrode and electrolyte of the following composition: 1.2 M LiPF_6_ in 3:7 ethylene carbonate:ethyl methyl carbonate (EC:EMC), with 10 wt % fluoroethylene carbonate (FEC) and 2 wt % vinylene carbonate (VC). After the initial conversion performed at C/20, cycling was continued with two cycles at C/10, two cycles at C/5, and 1000 cycles at C/2 in a voltage window between 50 mV and 1.0 V *vs* Li/Li^+^. C-rates were set individually for the different materials to 1200, 1500, 1900, and 1550 mA g^–1^ for SiN_0.90_-NP, SiN_0.63_-NP, SiN_0.39_-NP, and SiN_0.69_-μP, respectively. Such C-rate estimations were based on preliminary experiments. The corresponding electrochemical cycling results are represented in Figure 2a,b.

**Figure 3:**
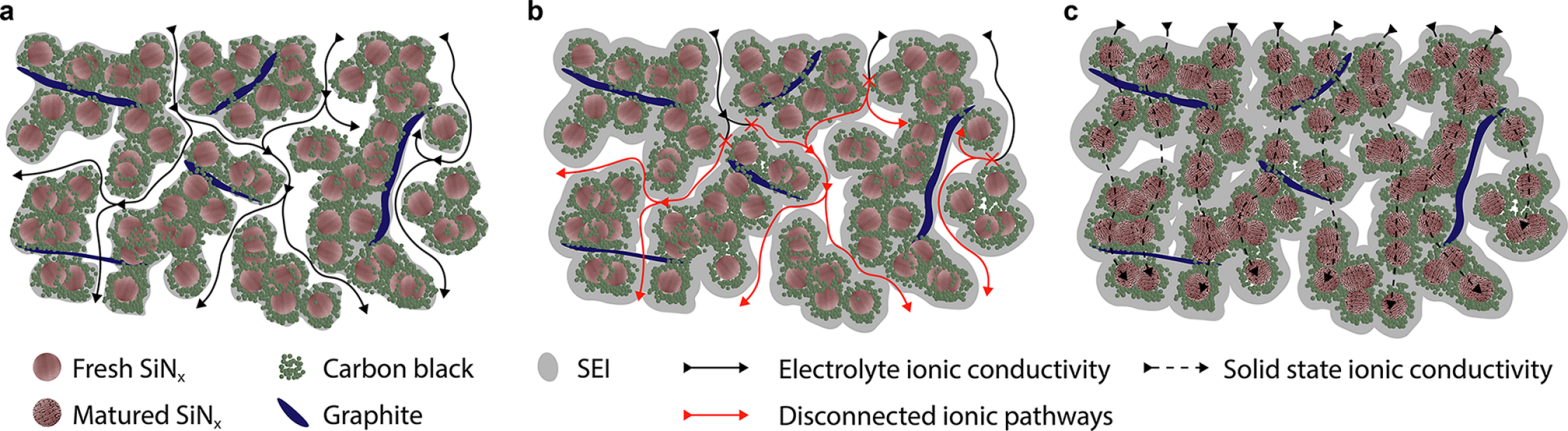
Schematic of lithium diffusion paths at the end of discharge in different stages of cycling: (a) after initial lithiation, thin SEI is formed, but pore structure and ionic conductivity is maintained at full lithiation; (b) in cycle 5–15, SEI gradually thickens to a point where the expanding particles block ionic conductivity through pores before reaching full lithiation; and (c) cycle 15 onward, electrolyte ionic conductivity is still prevented at high lithiation, but solid-state ionic conductivity through matured SiN_*x*_ particles facilitates continued operation.

The cells were cycled without a constant voltage step to emphasize the effect of any changes in the kinetic properties of the electrodes during cycling; consequently, the capacity drops as the rate is increased over the initial cycles. However, for all cells, the capacity continues to drop for 10–15 cycles even after the rate is stabilized at C/2. The continuous capacity *vs* voltage plot for the electrodes prepared from SiN_0.39_-NP (Figure 2d), where the effect of the capacity drop was the most pronounced, shows that this continued decrease of capacity is caused by limited lithiation of the electrode, while the degree of delithiation remains constant. From the differential capacity analysis (Figure 2e and Figure S5), the capacity loss is determined to be related to a decay exclusively in the low voltage activity of the electrode, as indicated by arrows in Figure 2e and, therefore, is originated from disappearance of highly lithiated α-Li_3.75_Si phase. Such suppression of α-Li_3.75_Si phase formation was previously observed for microcrystalline Si and was linked to the processes associated with SEI formation.^38^ Similarly, for SiN_*x*_, the gradual emergence of this effect and its selective impact above a certain degree of lithiation is hypothesized to be the result of gradual reduction of electrode porosity due to SEI formation in the initial cycles, limiting the space into which active particles can expand during lithiation. At a certain degree of lithiation, the particles fill up most of the available pore volume in the electrode at the expense of expelling electrolyte, thus reducing the electrode kinetics, which is heavily dependent on electrolyte ionic conductivity. As the porosity decreases in the early cycling due to SEI growth, this point is reached at gradually lower degrees of lithiation for every cycle, causing a gradual drop in available lithiation capacity. This process is schematically illustrated in Figure 3a,b. From the proposed mechanism, it also follows that the capacity drop should be more pronounced for materials with lower nitrogen content due to higher degree of expansion during lithiation and generally lower CE after formation (inset in Figure 2b), and should be independent of particle size, both of which are observed.

The above-mentioned capacity drop is only temporary and largely recovered during the subsequent cycling with the reemergence and stabilization of the low voltage activity, as seen in Figure 2f. Since the reduction in electrode porosity should be regarded as irreversible, the capacity recovery is, therefore, attributed to a gradual improvement in the Li^+^ diffusion properties of the electrode provided by changes in the SiN_*x*_ particles themselves. As mentioned above, the PDF analysis has confirmed the composition of the inactive matrix to be Li_2_SiN_2_, which has previously been shown to have excellent Li^+^ conductivity,^39^ on the scale of many solid electrolytes.^40^ It is likely that the segregation and formation of this ionically conductive matrix becomes more pronounced during cycling, the resulting solid-state ionic conductivity gradually making the electrode less dependent on porosity for intraelectrode ionic conductivity, as schematically shown in Figure 3c. This is in line with a previous study where SiN_*x*_ thin film electrodes were found to develop improved rate performance as a result of cycling.^29^ Therefore, it is reasonable to assume that additional structural transformations take place after the initial conversion reaction; however, the detailed study of such transformations of the Li_2_SiN_2_ matrix is beyond the scope of the present work.

After the initial capacity variation, the materials exhibit impressive cycling stability. Relative to the average capacity of the first three cycles at C/2, the electrodes fabricated from SiN_0.90_-NP, SiN_0.63_-NP, and SiN_0.39_-NP retain 103.4%, 87.3%, and 73.7% of their capacity after 1000 cycles. However, it should be noted that initial capacity and capacity at the end of cycling is the highest for SiN_0.39_-NP and lowest for SiN_0.39_-NP, with SiN_0.63_-NP in the middle. The samples with the lowest nitrogen content experience the biggest changes upon cycling making them somewhat similar to Si-based electrodes which experience a large drop in capacity after formation cycles.^36^ Interestingly, SiN_0.69_-μP exhibited only somewhat lower capacity retention than SiN_0.63_-NP, at 79.3%, despite consisting of larger, micron-sized particles, indirectly confirming the presence of the additional structural transformations taking place during long-term cycling, which leads to slow degradation of the material.

Conventional full cell testing is not deemed to be a suitable analysis at this point because of the low first cycle Coulombic efficiency. We did, however, conduct full cell tests with electrodes containing 60 wt % SiN_0.90_-NP and SiN_0.39_-NP that were partially prelithiated. The prelithiation was performed electrochemically using Li metal as a counter electrode prior to assemble of the full cell to mitigate the losses associated with the first cycle. Commercially available lithium iron phosphate (LFP) was used as a cathode for the testing in full cell. The results of such testing for both SiN_*x*_ materials are shown in Figure 2c. These results reflect the high Coulombic efficiency of SiN_0.90_-NP during long-term cycling in half cells (seen in the inset in Figure 2b), which in full cells retained 87.2% capacity after 250 cycles at C/2 in full cells, compared with 43.2% for SiN_0.39_-NP, relative to the average of the first three cycles at C/2.

### Evolution of SiN_*x*_ Nanomaterials during Cycling

**Figure 4:**
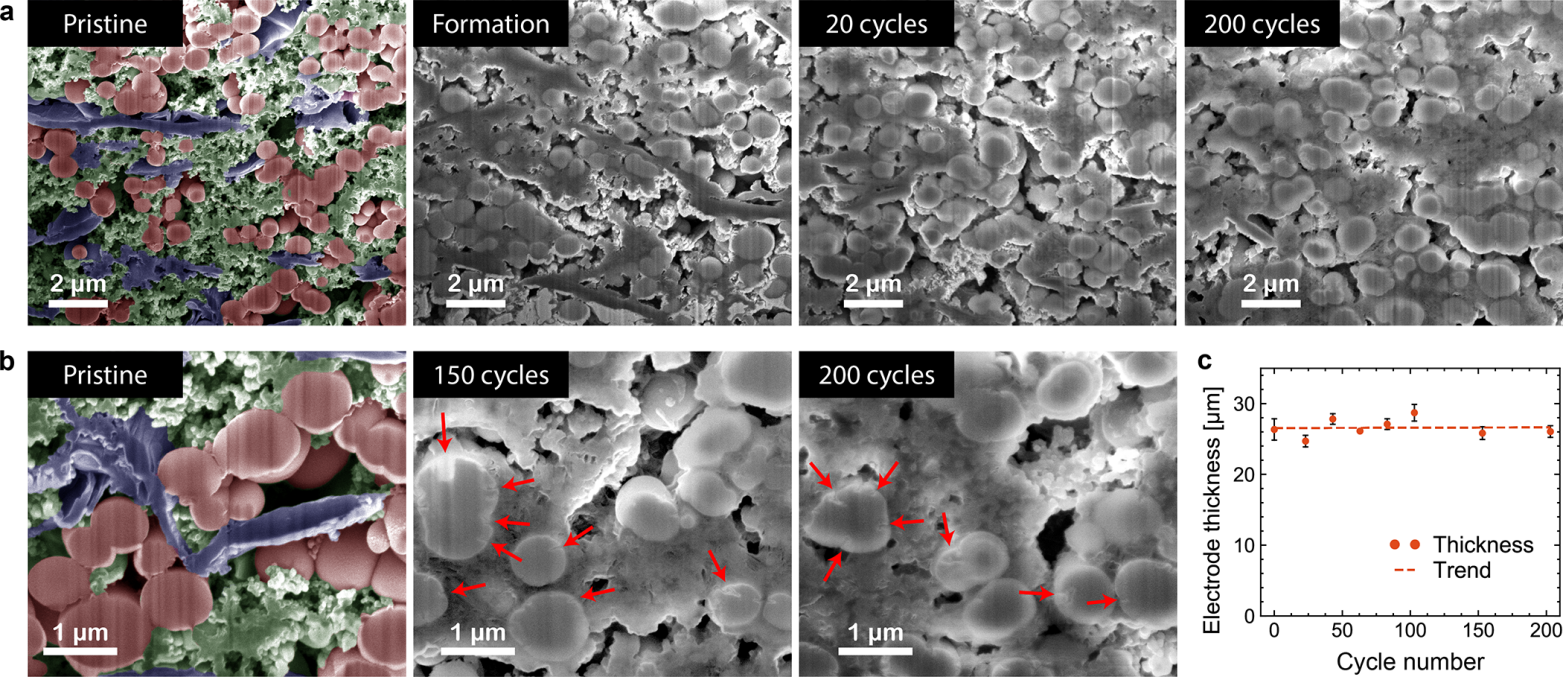
SEM images of FIB cross sections of electrodes containing 60 wt % SiN_0.69_-μP and evolution of electrode thickness at different stages of cycling: (a) low magnification of the electrode structure; and (b) high magnification showing structural changes in the particles (red arrows). The false color in the images of the pristine electrodes identifies the different electrode constituents - SiN_0.69_-μP (red), graphite (blue), and carbon black/binder (green). (c) Electrode thickness vs cycle number with the fitted linear trend (*t* = 0.0005·cycle +26.545 [μm], *r*^2^ = 0.0008). The error bars indicate the range of thickness measurements for each electrode.

To investigate the evolution of the materials and changes in the internal structure of electrodes during cycling, focused ion beam (FIB) cross sections coupled with microscopy analysis were performed on the electrodes in the pristine state, after formation cycles, and at several different points during subsequent 200 cycles. Figure 4a shows a selection of FIB-SEM images at different magnifications of the electrodes containing 60 wt % of SiN_0.69_-μPs at different stages of cycling, while additional images for all electrodes prepared using other compositions and sizes imaged at the same points of their lifetime are shown in Figures S6–S9. For clarity, the images of the pristine electrodes in Figure 4 have been false colored to identify the electrode constituents, where the SiN_0.69_-μPs are colored red, graphite flakes are blue, and the remaining components consisting of a binder and carbon-black is green. By comparing the FIB-SEM images prior to cycling and after formation cycles, it is evident that the electrodes experience some densification, that is, porosity loss due to SEI formation during the initial cycle as suggested above. This is most apparent in the smaller secondary pores, primarily in the areas containing high-surface-area carbon black, while the larger primary pore structure in the electrode is still preserved. Some further densification could be observed over the course of the following 20 cycles and is the most pronounced for the lower nitrogen content materials. After the initial densification, the electrode morphology is largely maintained up to 200 cycles.

Higher-magnification images for the SiN_0.69_-μP-based electrode (Figure 4b) show that some particles have developed minor surface cracks after 150 cycles as highlighted by red arrows on the image. However, they still retain most of their original morphology, as no further deterioration is evident in the following 50 cycles up to 200 cycles; the progress of these changes is determined to be slow and show no evidence of self-reinforcing behavior. This observation is in contrast with the changes typically observed for Si-based electrodes. For instance, Wetjen et al.^6^ have reported that pure Si particles undergo severe morphological changes during cycling under similar conditions after only 60 cycles: these changes include a transformation of Si from solid particles into porous networks of nanometer-sized branches, where electrodes containing 35 wt % of Si were found to irreversibly expand by more than 140% over the course of 60 cycles.^6^Figure 4c shows that the SiN_0.69_-μPs-based electrodes experienced almost negligible swelling over the course of 200 cycles as measured by SEM on the FIB cross sections of delithiated electrodes, emphasizing the stabilization effect achieved by *in situ* conversion reaction.

**Figure 5:**
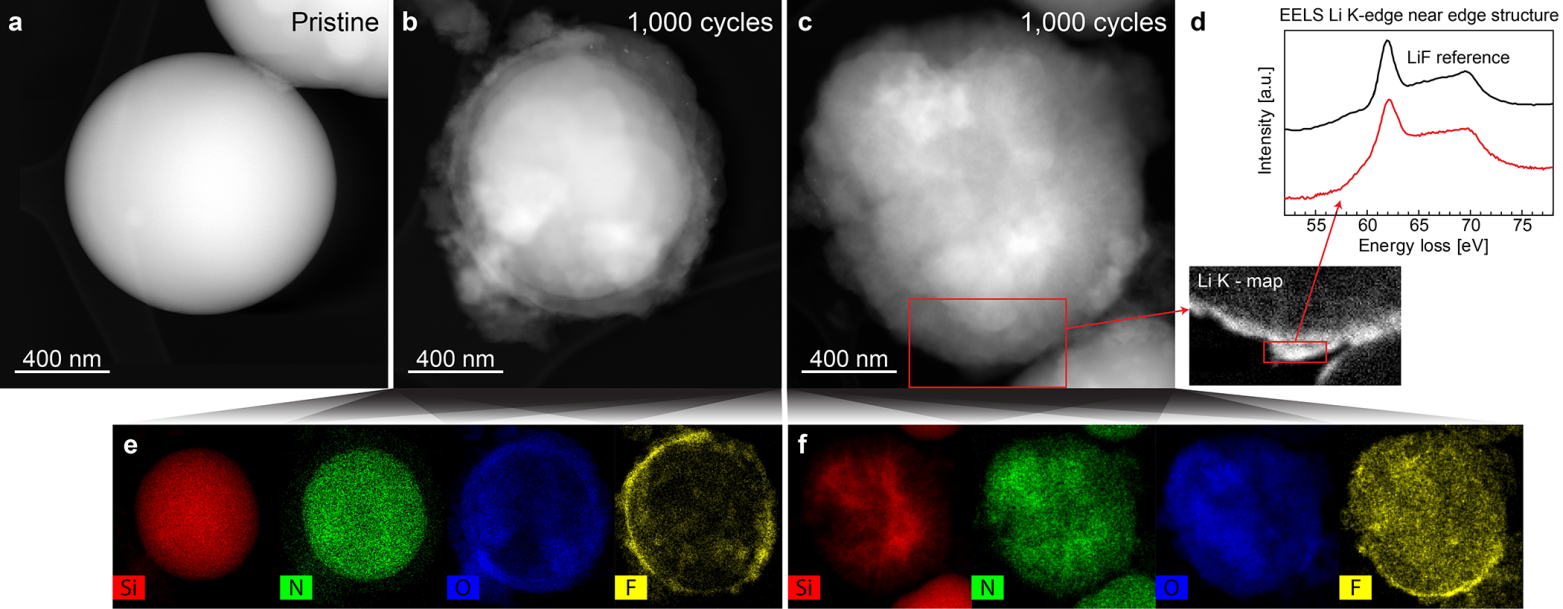
TEM post-mortem analysis of electrode material containing 60 wt % SiN_0.69_-μPs before and after 1000 cycles: (a) STEM-HAADF images of a pristine particle; (b, c) cycled particles; (d) EELS map of lithium and EELS near edge structure of the SEI, the EELS reference spectrum for LiF is from Wang et al.;^43^ (e, f) EDS maps of silicon, nitrogen, oxygen and fluorine in the cycled particles.

A further degradation study of SiN_*x*_ materials was performed by TEM post-mortem analysis of SiN_0.69_-μPs after 1000 cycles performed at a rate of C/2. This analysis revealed that a majority of the SiN_0.69_-μPs underwent only minor morphological changes over the course of these 1000 cycles, as exemplified by the STEM images in Figure 5a,b, showing SiN_0.69_-μPs particles before and after cycling, respectively. Corresponding EDS maps of the cycled particle (Figure 5e) show no significant changes in the elemental distribution within these particles, with the exception of a thin, fluorine-rich SEI layer, and an oxide shell primarily attributed to air exposure during sample transfer. The expected segregation into of active Si domains and Li_2_SiN_2_ matrix was not distinguishable in transmission, which is most likely due to the size of the Si domains relative to the particle size (the work conducted for SiO_*x*_ suggested that size of the Si domains is at nm scale^27^). Some SiN_0.69_-μPs particles were found to be more affected by cycling and experienced some elemental segregation, as seen in the image and EDS maps in panels c and f, respectively, of Figures 5. At the present moment, no specific reason for slight variations of behavior was identified, although varying particle size and nitrogen content are the likely factors.

Lithium mapping by electron energy loss spectroscopy (EELS) revealed that the SiN_0.69_-μPs particles retain a dense but relatively thin SEI layer after 1000 cycles, as could be seen in Figure 5d. Furthermore, LiF was determined as the primary component of SEI layer using EELS near-edge structure. A similar scenario was observed for the SiN_*x*_ particles which were more affected by degradation, therefore, indicating that the changes in SiN_*x*_ particles during cycling are mainly related to an internal redistribution of elements, rather than the typical surface-forming degradation as observed for pure Si.^6^ It should be noted that in cycling experiments conducted for the present work, FEC was used as electrolyte additive due to its ability to improve the cyclability through aiding in the formation of a LiF-rich SEI layer.^41^ FEC has, however, been reported to be continuously consumed on both Si-based electrodes and lithium counter electrodes, when FEC is consumed, capacity of an electrode rapidly fades.^42^ However, the stable SEI layer on SiN_0.69_-μP is responsible for long-lasting performance and relatively constant capacity over the large number of cycles. This is in agreement with the observed high and stable Coulombic efficiency during extended cycling of SiN_*x*_ containing electrodes (Figure 2b). It must be reiterated, that the materials exhibit unsatisfactory first-cycle Coulombic efficiency, which could be remediable by carbon coating, as has been reported in a previous study,^34^ heat treatment, and tuning of the nitrogen content, which should be a focus of the further studies of this material.

### SiN_*x*_ as an Addition to Graphite Electrodes

**Figure 6:**
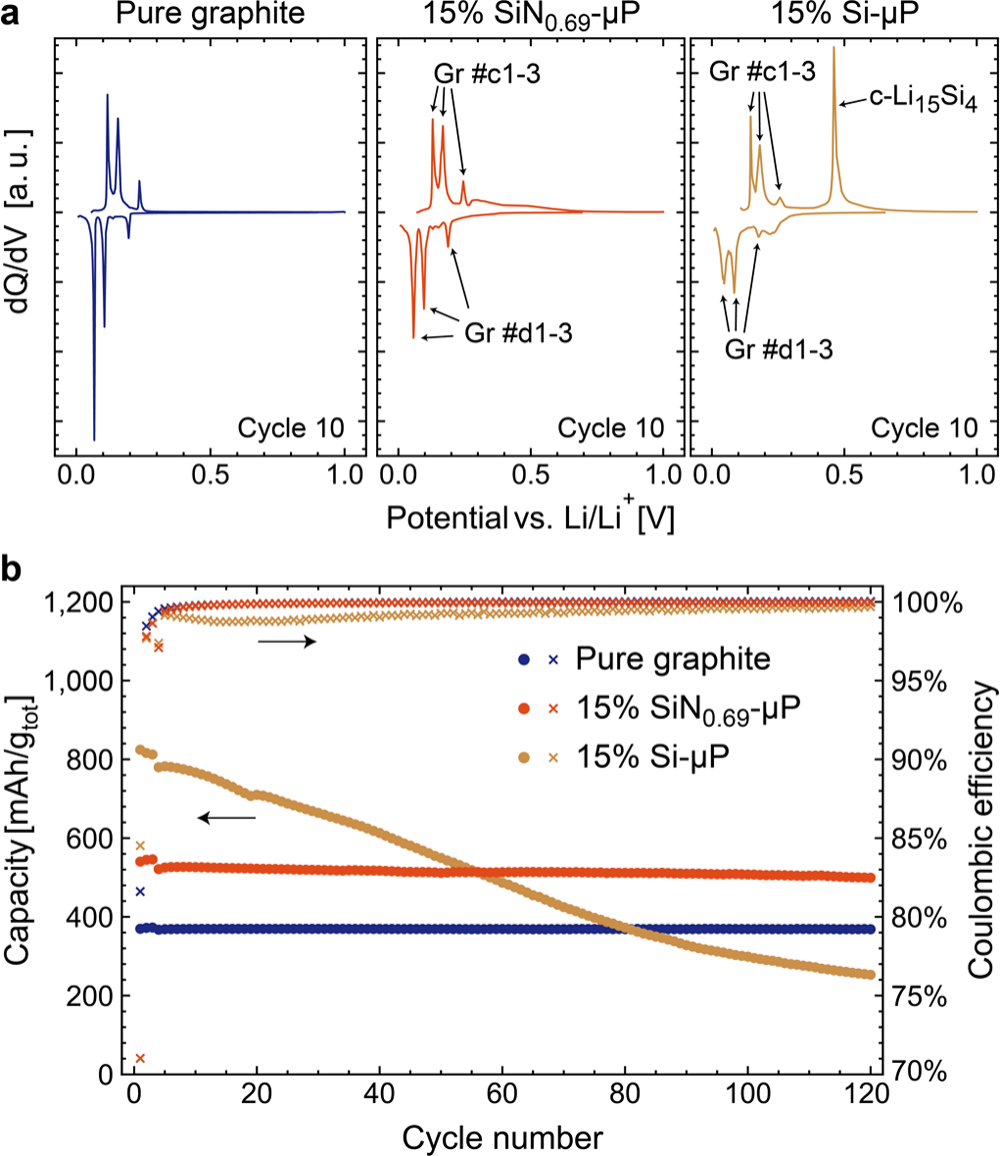
Electrochemical analysis of SiN_*x*_ in predominantly graphitic electrodes cycled to 5 mV *vs* Li/Li^+^: (a) differential capacity in the 10th cycle of a pure graphitic electrode and electrodes containing 15% pure Si and SiN_0.69_-NP, showing the formation of c-Li_15_Si_4_ only in the pure Si containing electrode; (b) a comparison of the cycling stability of the same electrodes.

Currently, the use of the Si-based materials in state-of-the-art LIBs is limited to low Si content in anodes primarily consisting of graphite.^44^ The small amount of Si is used to slightly “boost” the capacity of such anodes, but in order to minimize degradation, the amount of Si has to be rather low. Therefore, the compatibility of SiN_*x*_ as an “additive” to graphitic electrodes was investigated in the present work by comparing the performance of three electrodes: one containing 15 wt % SiN_0.69_-μP, one containing 15 wt % pure crystalline-Si of comparable particle size (Si-μP), and a pure graphite reference. All three types of electrodes were cycled to 5 mV *vs* Li/Li^+^ (the details of cycling and preparation are described in SI) and analyzed from the perspectives of cycling stability and mechanism. Figure 6a shows a comparison of the differential capacity analysis of these electrodes at cycle 10. Because of a low content of Si (or SiN_0.69_-μP), graphite peaks are clearly seen for all electrodes. However, the characteristic sharp peak at 430 mV corresponding to Li_15_Si_4_ phase delithiation dominates the delithiation curve for the Si-μP containing electrode. Such behavior is expected for Si lithiated below approximately 50 mV.^45^ However, as crystalline-Li_15_Si_4_ (c-Li_15_Si_4_) is regarded as detrimental to the cyclability of the material,^46^ it is notably not present in the SiN_0.69_-μP containing electrode. This additionally demonstrates that the formation of c-Li_15_Si_4_ has been largely suppressed in SiN_*x*_ materials, despite the domains of pure Si being likely the active part of the SiN_*x*_ material after formation cycles. This suppression is in agreement with the observations from the PDF analysis, as discussed above, and attributed to a clamping (or buffering) effect of the matrix surrounding the active Si domains. Such confinement limits the degree of lithiation and structural changes necessary to form c-Li_15_Si_4_, as has previously been reported for tightly bound Si thin films,^47^ as well as hypothesized as a stabilization mechanism for SiO_*x*_-based electrodes.^27^

The continued cycling of these electrodes (Figure 6b) further demonstrates the excellent cycling stability of SiN_0.69_-μP compared with Si-μP, as well as a continuously high CE after formation cycles. Notably, the electrode containing Si-μP degrades below the capacity expected from the graphite content alone, demonstrating that the Si degradation is also detrimental to the performance of the complete graphite-based electrode. This is likely due to densification of the porous electrode by continuous formation of SEI, as seen from the low Coulombic efficiency during cycling (Figure 6b)—an issue that was not observed for the SiN_0.69_-μP-containing electrodes.

## Conclusions

In the present work, amorphous SiN_*x*_ nanoparticles were synthesized as promising materials for the future LIB anodes: their lithium storage capacity is tunable through the adjustment of their chemical composition. These SiN_*x*_ nanoparticles, whose stoichiometry and particle’ size could be controlled by synthetic conditions, were found to exhibit excellent cycling stability over more than 1000 cycles. While PDF analysis was deployed to confirm the mechanism of SiN_*x*_ functionality, the post-mortem FIB-SEM and TEM analyses have illustrated that the exceptional cycling stability of this class of materials stems from the following two primary properties:

Stability of the material at the particle level during electrochemical cycling originating from the *in situ* conversion reaction. That results in the formation of the inactive matrix limiting the pulverization and fracturing of Si active domains, while at the same time providing high intraparticle Li^+^ conductivity.
Stable material’s surface, allowing formation of a robust SEI layer on the intrinsic surface area that is maintained for a large number of cycles.

Together, these properties effectively mitigate the two major degradation mechanisms of Si-based electrodes–material disintegration and uncontrolled SEI growth. Additionally, the suppression of the c-Li_15_Si_4_ phase formation usually seen during deep lithiation of pure Si^47^ is expected to further stabilize the material and makes it a suitable material for use in conjunction with graphite in modern LIBs.

## Experimental Section

### Materials

All chemicals were used as received, without further purifications. Silane (SiH_4_, ultrahigh purity) and Ar (ultrahigh purity) were purchased from Praxair. Ammonia gas (NH_3_, ultrahigh purity) was purchased from Nippon. Sodium carboxymethyl cellulose (CMC, Mw = 90000), poly(acrylic acid) (PAA) and potassium hydroxide were purchased from Sigma-Aldrich. Citric acid was purchased from Merck. Graphite(C-NERGY KS6L) and carbon black (C-NERGY Super C65) were purchased from Imerys.

### Synthesis of SiN_*x*_ Particles and Nanoparticles

The general synthesis of amorphous Si nanoparticles through pyrolysis of SiH_4_ in hot wall free space CVD reactor was described elsewhere.^36^ In the present work, amorphous SiN_*x*_ particles were synthesized using a similar procedure modified by introducing NH_3_ as nitrogen-containing precursor into reactor. The samples described herein were synthesized with SiH_4_:NH_3_ flow rate ratios ranging from 1:1 to 3:1, and pyrolysis temperatures ranging from 600 to 650 °C. The smaller particles (SiN_0.39_-NP, SiN_0.63_-NP, SiN_0.90_-NP) were prepared by diluting the precursors with Ar gas in a 1:2 (SiH_4_+NH_3_:Ar) flow rate ratio, whereas the larger SiN_0.69_-NP particles were made without Ar dilution. Nanoparticles were collected by filtering downstream and stored in an Ar-filled glovebox with <0.1 ppm of O_2_ and <0.1 ppm of H_2_O levels.

### Electrochemical Testing

For all composition of SiN_*x*_, the electrodes containing 60 wt % of the SiN_*x*_ particles were prepared by making a slurry consisting of the active SiN_*x*_ particles, graphite, carbon black, and CMC binder in a 60:15:10:15 ratio by mass. Electrodes containing 15 wt % Si or SiN_*x*_ were made with SLP30 (TIMCAL) graphite, carbon black, and PAA binder in a 15:75:5:5 ratio by mass. All slurries were prepared using a buffer solution at pH 3 as solvent, made from 0.173 M citric acid and 0.074 M KOH in deionized water, which has been found to improve the cycle life of CMC/Si-based electrodes.^48^ The slurries were thoroughly mixed in a planetary centrifugal mixer (ARE-250, Thinky), and electrodes were casted on 16 μm deoxidized and surface structured copper foil (SE-Cu58, Schlenk) using a tape caster (MC-20, Hohsen) equipped with a Baker type applicator. The electrodes were dried in ambient conditions for 24 h, followed by 3 h of additional drying at 120 °C under vacuum in a glovebox antechamber oven.

For the fabrication of batteries, the disks were cut from the dried electrodes using a 15 mm diameter disc cutter (Hohsen) and mounted in 2032 stainless steel coin cells (Hohsen). Half cells were made with a 15 mm diameter lithium metal counter electrode (99.99%, LinYi Gelon LIB Co.), an 18 mm diameter microporous polypropylene separator (Celgard 2400), and an 18 mm diameter glass fiber separator (Viledon 2207-25), in that order. The electrolyte was supplied by Solvionic and consisted of 1.2 M LiPF_6_ in 3:7 ethylene carbonate:ethyl methyl carbonate (EC:EMC), with 10 wt % fluoroethylene carbonate (FEC) and 2 wt % vinylene carbonate (VC). Cell fabrication was conducted in an Ar-filled glovebox with <0.1 ppm of O_2_ and <0.1 ppm of H_2_O.

To allow for long-term testing of the materials, half cells used for long-term cycling (Figure 2a) were made with low areal capacity in the range 0.32–0.47 mAh/cm^2^, as dendrite formation is limited at low current density on the lithium counter electrode.^49^ Formation of these cells consisted of two cycles at C/20 with C/50, C/100, and C/200 taper steps during lithiation, to ensure completion of the conversion reaction. The conversion reaction has previously been found to require slow lithiation to prevent premature cutoff caused by the overpotential related to nucleation of the new phase constituents.^29^ Delithiation, not requiring the same slow rate, was simply conducted at a rate of C/20 to 1.0 V *vs* Li/Li^+^ with no taper. This was followed by two cycles at C/10 and two cycles at C/5, before continuing with 1000 cycles at C/2, all between 50 mV and 1.0 V *vs* Li/Li^+^, with no constant voltage or taper steps.

Full cells were assembled using commercial 1 mAh/cm^2^ LFP cathodes (Customcells), a single polypropylene separator (Celgard 2400), and otherwise in the same manner as the half cells. Cycling was conducted between 2.4 and 3.4 V *vs* Li/Li^+^, starting with two cycles each at C/10 and C/5, and 200 cycles at C/2. The SiN_*x*_ containing electrodes used in full cells were first partially prelithiated in a half cell by lithiation to 50 mV *vs* Li/Li^+^ at a rate of C/20 with C/50, C/100 and C/200 taper steps, and delithiation to 1.0 V *vs* Li/Li^+^ at a rate of C/20, to account for the Li loss associated with the conversion reaction.

For FIB/SEM analysis (Figure 4), thicker electrodes with an areal capacity in the range of 1.68–1.85 mAh/cm^2^ were used to better facilitate analysis of electrode densification and thickness changes during cycling. These electrodes were cycled between 50 mV and 1.0 V *vs* Li/Li^+^ with a formation procedure consisting of three cycles at C/20 with C/50 taper step on lithiation, before cycling at a predetermined number of cycles at C/4 before being disassembled for analysis.

Testing of Si and SiN_*x*_ in predominantly graphitic electrodes (Figure 6) was conducted with a higher areal capacity, in the range of 2.08–2.55 mAh/cm^2^, to illuminate the differences between pure Si and SiN_*x*_ when used as an “additive” to commercial graphitic electrodes. These were cycled with a test schedule tailored for graphite, between 5 mV and 1.0 V *vs* Li/Li^+^, with a formation consisting of 3 cycles at C/20 with a 1 h constant voltage step at 5 mV on lithiation, followed by 120 cycles at C/5, also with a 1 h constant voltage step at 5 mV on lithiation.

All cycling experiments were conducted at 25 °C in temperature-controlled cabinets (VWR INCU-Line) using an Arbin LBT battery tester.

### Microscopy Characterization

SEM analysis of the pristine materials was conducted using a Hitachi S-4800 instrument with a field emission gun operated at 30 kV, and images were acquired using a secondary electron (SE) detector. The instrument is equipped with low-voltage TEM capability, and samples were therefore prepared in the same manner as the pristine material TEM samples described in the previous section. Primary particle size distribution measurements were made by manual analysis of SEM images using ImageJ software.

TEM samples of pristine materials were prepared by first dispersing a small amount of powder in ethanol, aided by ultrasonication for 5 min. A drop of the dispersion was placed on a holey carbon TEM grid (300 mesh copper, EM Resolutions) with holey carbon film and dried. Before inserting into the TEM, the samples were plasma cleaned for 30 s in 25 at% O_2_ in Argon (Model 1020 Plasma Cleaner, Fischione Instruments) to remove organic contamination, using a shielded specimen holder to protect the amorphous holey carbon film.

For post-mortem TEM analysis, the cell was first delithiated to 50 mV *vs* Li/Li^+^ at a rate of C/20 before being disassembled in an Ar-filled glovebox and the anode extracted. To prevent salt deposition from drying electrolyte, the electrode was first soaked in pure DMC (Sigma-Aldrich) for 5 min, and then carefully rinsed under running DMC (2 mL in total) from a syringe before being thoroughly dried under vacuum. Using a scalpel, electrode material was carefully scraped form the current collector directly onto a holey carbon TEM grid. The grid was tapped to remove any excess materials before being sealed under Argon, and only opened just prior to insertion into the TEM.

All TEM analysis was conducted using an FEI Titan G2 60–300 microscope equipped with a Wien-filter monochromator and DCOR Cs probe corrector. The instrument was operated in scanning TEM (STEM) mode with an acceleration voltage of 300 kV, a nominal probe current of 80 pA and a probe convergence angle of 30.8 mrad. Under such operation, the nominal spatial resolution of the instrument is rated to 0.8 Å. Images were primarily acquired using bright field (BF) and high angle annular dark field (HAADF) STEM detectors with collection angles from 0 to 6.3 mrad and 58.5–200 mrad, respectively.

EELS spectra were acquired using a Gatan GIF Quantum ER 965 spectrometer with Ultrafast DualEELS, using a dispersion of 0.1 eV/channel and collection angle of 33.1 mrad. The spectrum image in Figure 5f was acquired with a resolution of 128 × 96 pixels, with the energy loss spectrum in each pixel covering a range from approximately −20 to 185 eV. The fwhm energy resolution in vacuum during acquisition was measured to be 1.1 eV. The Li K-edge map and spectrum in Figure 5f was extracted from the indicated region of the SEI shown in the same figure, and the background was subtracted using a standard power law background fitted to the region from 47.55 to 53.25 eV, just before the expected onset of the Li K-edge.

Energy dispersive spectroscopy (EDS) was conducted using a windowless four-detector SuperX EDS system. Maps were acquired from pristine and cycled materials, and elemental quantification was done using the Cliff-Lorimer (k-factor) method as implemented in the Bruker Esprit software.

### FIB Cross Sections

FIB/SEM cross section fabrication and analysis was done using an FEI Helios NanoLab DualBeam FIB instrument, using a 30 kV Ga ion source. For cross section preparation, a protective Pt layer was first deposited on the electrode surface, followed by coarse milling through the entire electrode with a beam current of 21 nA at 30 kV and cross section cleaning at gradually lower current, finishing at 0.28 nA. Images were acquired using the electron beam at 5 kV and ICE detector (secondary electron/secondary ion). Pristine electrodes were analyzed as-is, while cycled electrodes were washed in DMC in the manner described for post-mortem TEM samples and only exposed to air just prior to insertion into the instrument.

### Pair Distribution Function

The samples for PDF analysis were prepared inside an argon filled glovebox with <0.1 ppm of O_2_ and <0.1 ppm of H_2_O. At first, cycled electrodes were washed in the same manner as for TEM sample preparation, and material was then scraped off the current collector and put in capillaries which were sealed inside the glovebox. Bragg diffraction patterns were acquired at the I15–1 beamline at Diamond Light Source (76 keV beam energy) using a 2D detector (PerkinElmer XRD 1611 CP3). Initial processing was conducted using the Gudrun software,^50^ while fitting of PDF data was conducted using the PDFgui software^51^ and was refined based on published structural data of the relevant phases: c-Si,^52^ graphite,^53^ LiC_6_,^54^ LiC_12_,^55^ α-Si_3_N_4_,^56^ c-Li_3_N,^57^ c-LiSi_2_N_3_,^58^ c-Li_2_SiN_2_,^59^ c-Li_7_Si_3_,^60^ c-Li_13_Si_4_,^61^ and c-Li_15_Si_4_.^62^ No attempt was made to subtract the PDF signal from the carbon-containing species, which were rather fit as parts of the sample.

## Supporting Information Available

SEM images of SiN_*x*_ nanoparticles, XRD characterization, pair distribution function data, details of calculations of delithiation capacities, differential capacities during long-term cycling, port-mortem FIB-SEM analysis (PDF)
