# Supplementary material for: Stoichiometry-Controlled Reversible Lithiation Capacity in Nanostructured Silicon Nitrides Enabled by in Situ Conversion Reaction
Source: ACS Nano. 2021 Sep 27;15(10):16777–87. doi: 10.1021/acsnano.1c06927 (PMC8552487; doi:10.1021/acsnano.1c06927)
Supplement: Supplementary file 1 — nn1c06927_si_001.pdf [file nn1c06927_si_001.pdf]

Supporting information.

Stoichiometry-Controlled Reversible Lithiation Capacity in Nanostructured Silicon Nitrides Enabled by *in Situ* Conversion Reaction.

Asbjørn Ulvestad[a], Marte O. Skare[a], Carl Erik Foss[a], Henrik Krogsæter[a,b], Jakob F. Reichstein[a,†], Thomas J. Preston[a], Jan Petter Mæhlen[a], Hanne F. Andersen[a], Alexey Y. Koposov[a,c]**

AUTHOR ADDRESS.

[a] Department of Battery Technology, Institute for Energy Technology, Instituttveien 18, NO-2027 Kjeller, Norway

[b] Department of Materials Science and Engineering, Norwegian University of Science and Technology, Alfred Getz vei 2, NO-7491 Trondheim, Norway

[c] Center for Materials Science and Nanotechnology, Department of Chemistry, University of Oslo, P.O. Box 1033, Blindern, 0371 Oslo, Norway

Pristine materials characterization

SEM images of pristine SiN_x particles

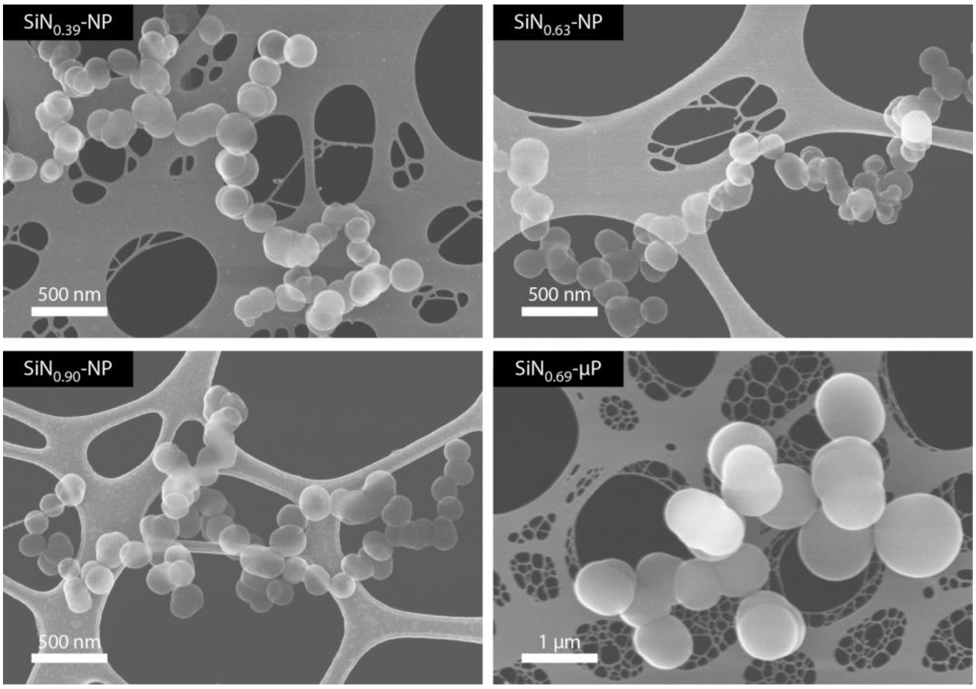

Figure S1. SEM images of the pristine nanoparticles of all samples studied in the present work. Note the different scale bar for the larger $SiN_{0.69}$-μP.

Distribution of elements in SiN_x particles.

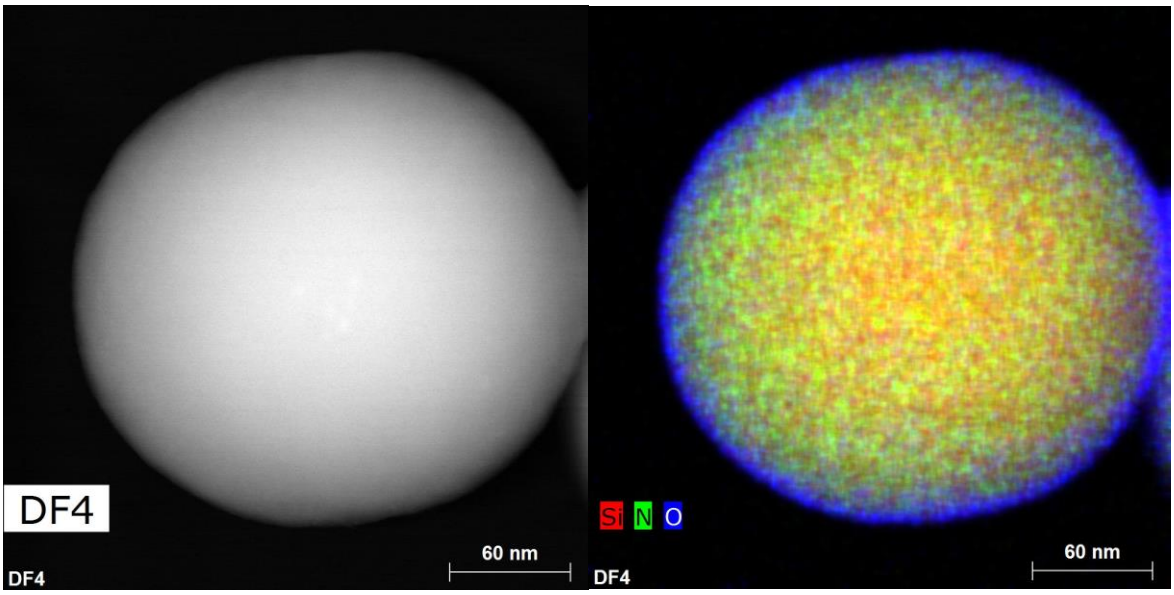

Figure S2. Representative TEM-EDS mapping of pristine SiNx nanoparticle demonstrating uniform distribution of Si and N as well as formation of native oxide layer.

X-ray diffraction of pristine materials

X-ray diffraction analyses of the pristine powders were carried out using a Bruker AXS D8 Advance diffractometer with a Cu Kα X-ray source (λ = 1.5406 Å), LynxEye detector and flat plate sample holder. Diffractograms were acquired in a 2Θ range from 15° to 70°, with a step size of 0.0195°, and are shown in Figure S3 with a diffractogram from a pure amorphous Si (a-Si) reference.

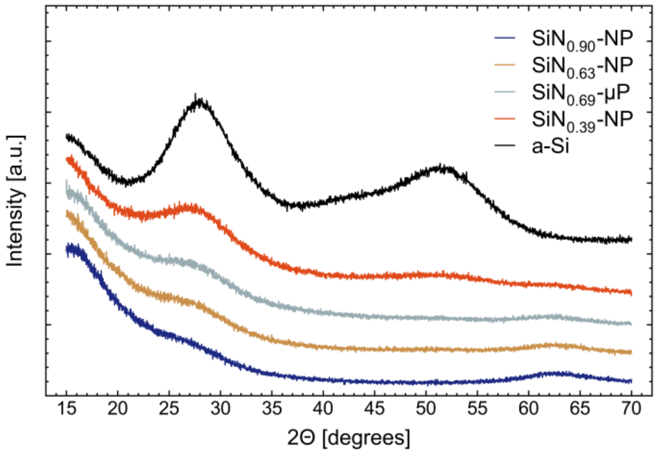

Figure S3. X-ray diffraction patterns for the pristine $SiN_{0.90}$-NP, $SiN_{0.63}$-NP, $SiN_{0.69}$-µP and $SiN_{0.39}$-NP, as well as a pure a-Si reference.

Pair distribution function (PDF) data

Figure S4 (panel A) shows the full PDFs of different samples as acquired and analyzed as described in the methods section in the main paper. A subset of the short-range correlations of the same data can be seen in Figure S4 (panel B), with annotations identifying correlations related to notable peaks.

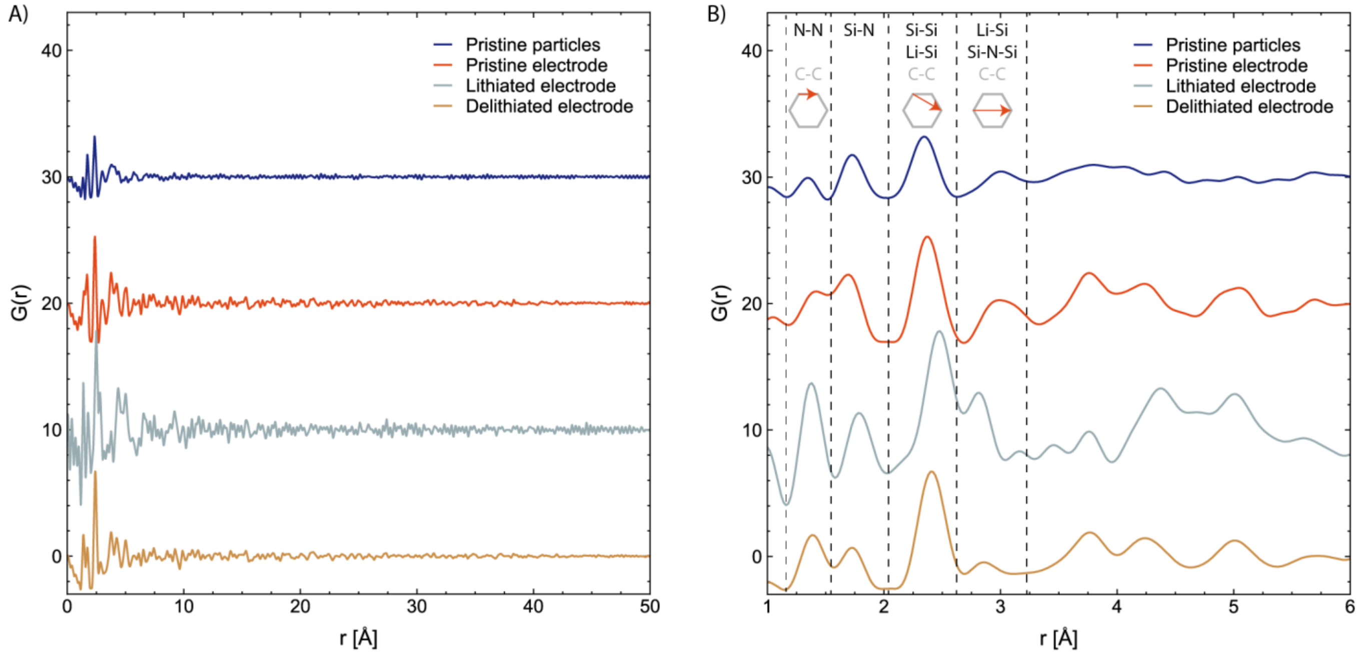

Figure S4. Pair distribution functions of electrode material containing 60 wt.% $SiN_{0.63}$-NP in the pristine state, after the initial lithiation and after the subsequent delithiation, as well as only the $SiN_{0.63}$-NP particles. Showing the full range (a), and the expanded view of the short-range (b) correlations with some relevant near bond distances of the relevant elements determined from c-Si,[1] graphite,[2] α-Si_3N_4,[3] Li_2SiN_2,[4] c-Li_7Si_3,[5] c-$Li_{13}Si_4$,[6] and c-$Li_{15}Si_4$.[7] The hexagon schematics illustrate the relevant carbon-carbon bond correlations originating from the graphite, LiC_6 and LiC_{12}.

Calculation of delithiation capacity and Coulombic efficiency from conversion reaction

For the calculation of specific reversible capacity and bulk Coulombic efficiency of the studied materials we employed the methods derived earlier.[8] Here, $Li_{3.5}Si$ was used as the composition of the fully lithiated Si, rather than the conventionally used $Li_{3.75}Si$, assuming complete lithiation is unlikely to happen when using a lithiation cut-off at 0.05V *vs.* Li^+/Li, which, as shown before,[9] largely avoids the formation of c-$Li_{3.75}Si$. The specific bulk reversible and irreversible capacities can be calculated using the equations:[8]

$$Cap_{R,bulk,specific} = \frac{(1 - k_1 x)\, 3.5F}{M_{Si} + xM_N}$$

$$Cap_{I,bulk,specific} = \frac{k_2 x\, F}{M_{Si} + xM_N}$$

Where F is Faraday's constant, M_{Si} and M_N are the molar masses of silicon and nitrogen, and x is the nitrogen to silicon ratio in the initial SiN_x, and k_1 and k_2 are the ratios of silicon to nitrogen and lithium to nitrogen in the matrix phase, respectively, *i.e.* 0.5 and 1.0 for Li_2SiN_2. The bulk Coulombic efficiency is then given by:[8]

$$CE_{C,bulk} = \frac{1}{\frac{k_2 x}{3.5(1 - k_1 x)} + 1}$$

Using k_1=0.5, k_2=1.0, and the compositions of $SiN_{0.90}$-NP, $SiN_{0.63}$-NP, $SiN_{0.39}$-NP, and $SiN_{0.69}$-µP, this results in reversible capacities of 1265 mAh g^{-1}, 1738 mAh g^{-1}, 2245 mAh g^{-1}, and 1623 mAh g^{-1}, respectively, and respective Coulombic efficiencies of 68%, 79%, 88% and 77% as reported in the main text.

Differential capacity analysis during long term cycling

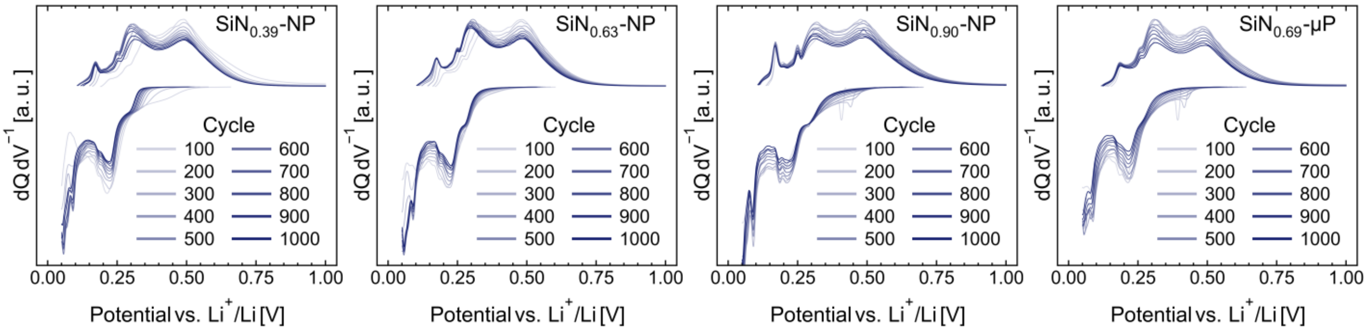

Figure S5. Differential capacity analysis of all samples from the cycling data presented in Figure 2 within the main text of the article.

FIB post-mortem images of all samples

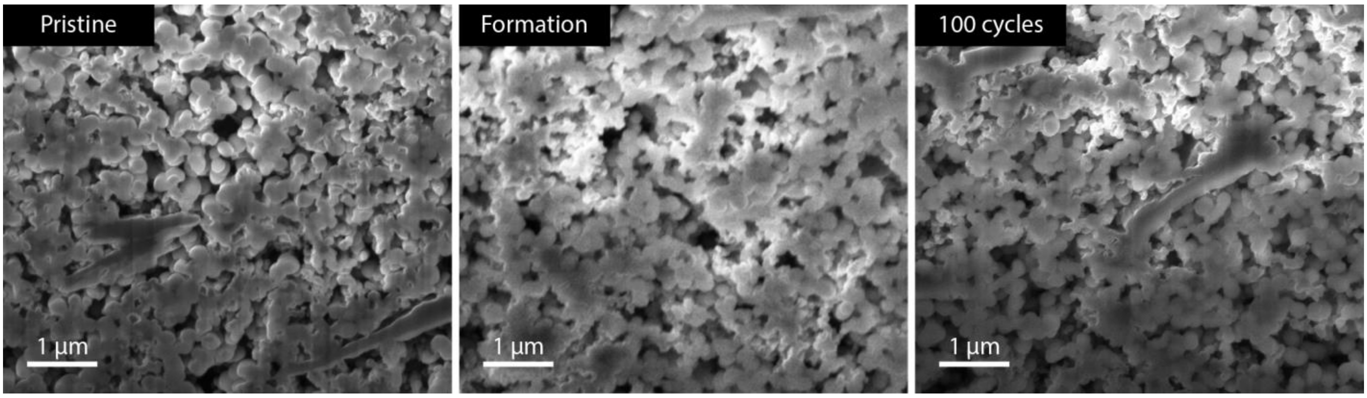

Figure S6. SEM images of FIB cross sections made from electrodes containing 60 wt. % $SiN_{0.90}$-NPs before cycling, after formation and after 100 subsequent cycles (from left to right).

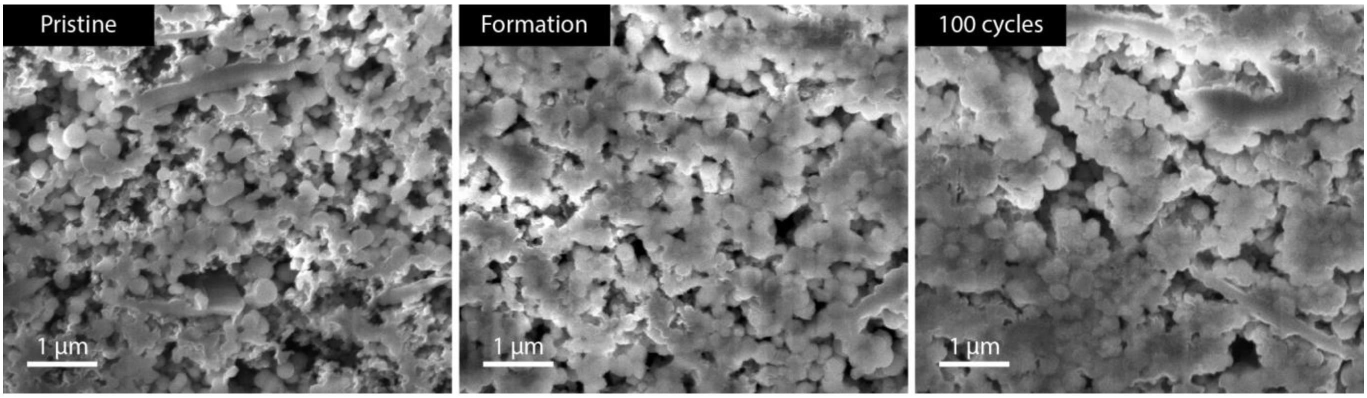

Figure S7. SEM images of FIB cross sections made from electrodes containing 60 wt. % $SiN_{0.63}$-NPs before cycling, after formation and after 100 subsequent cycles (from left to right).

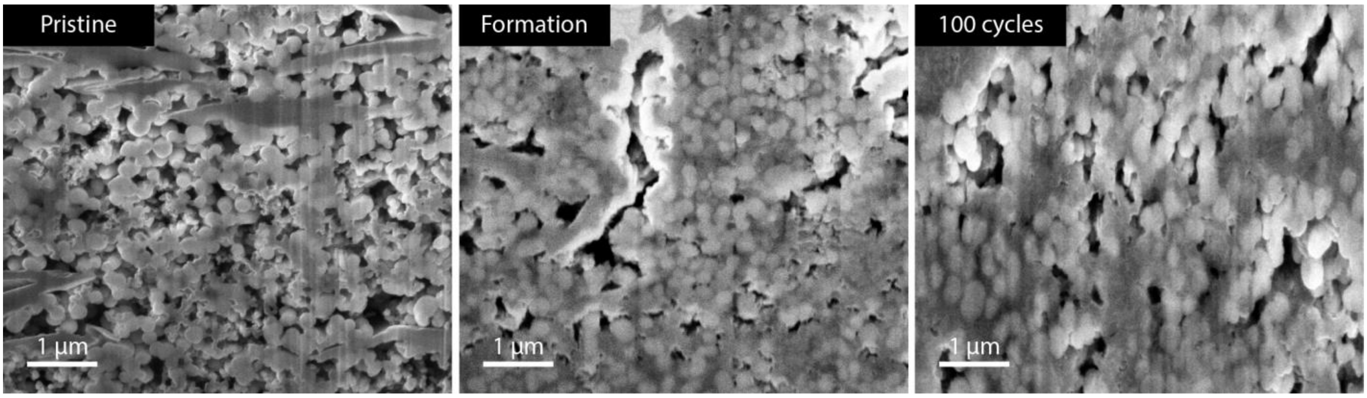

Figure S8. SEM images of FIB cross sections made from electrodes containing 60 wt. % $SiN_{0.39}$-NPs before cycling, after formation and after 100 subsequent cycles (from left to right).

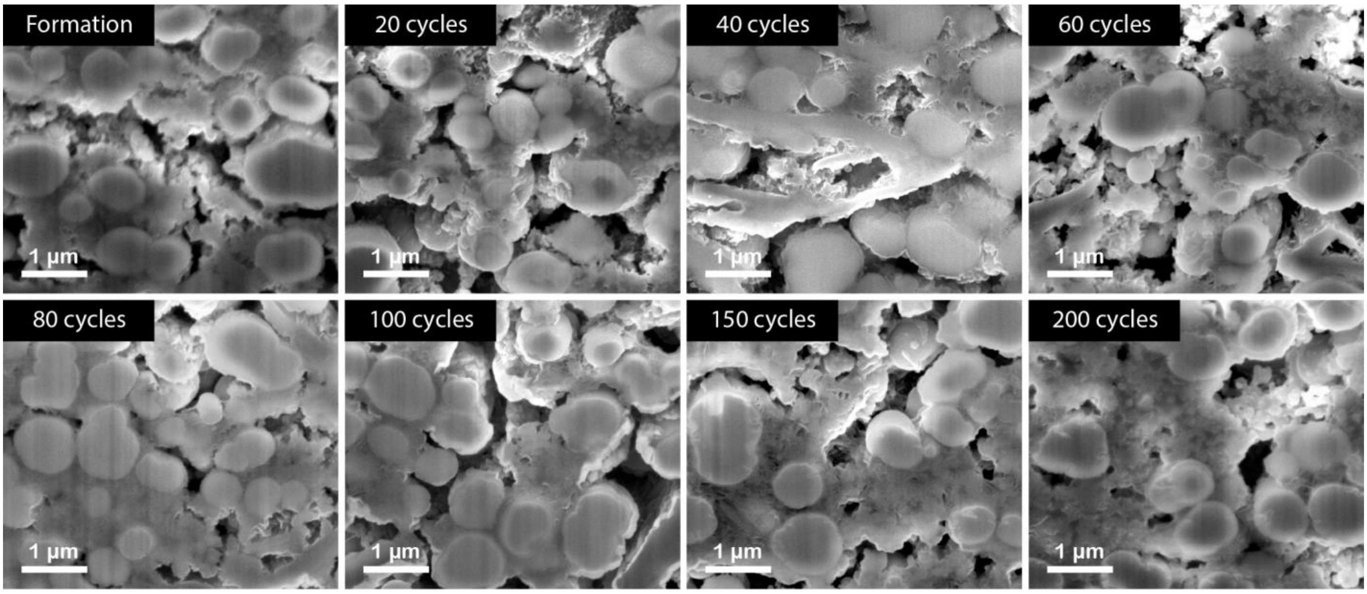

Figure S9. SEM images of FIB cross sections made from electrodes containing 60 wt. % $SiN_{0.69}$-µPs before cycling, after formation and after 20, 40, 60, 80, 100, 150 and 200 subsequent cycles.

References

1 Yim, W. M. & Paff, R. J. Thermal Expansion of AlN, Sapphire, and Silicon. *J. Appl. Phys.* ***45**, 1456-1457 (1974).*

2 Lei, L., Yin, W., Jiang, X., Lin, S. & He, D. Synthetic Route to Metal Nitrides: High-Pressure Solid-State Metathesis Reaction. *Inorg. Chem.* ***52**, 13356-13362 (2013).*

3 Billy, M., Labbe, J.-C., Selvaraj, A. & Roult, G. Modifications Structurales du Nitrure de Silicium en Fonction de la Temperature. *Mater. Res. Bull.* ***18**, 921-934 (1983).*

4 Casas-Cabanas, M., Santner, H. & Palacín, M. R. The Li–Si–(O)–N System Revisited: Structural Characterization of Li21Si3N11 and Li7SiN3O. *J. Solid State Chem.* ***213**, 152-157 (2014).*

5 Schnering, H.-G., Nesper, R. v., Curda, J. & Tebbe, K.-F. Structure and Properties of Li14Si6/Li/2.33/Si/, the Violet Phase in the Lithium-Silicon System. *Z. Metallk.* ***71**, 357-363 (1980).*

6 Schäfer, H., Axel, H., Menges, E. & Weiss, A. Zur Kenntnis des Systems Lithium—Silicium. *Zeitschrift für Naturforschung B* ***20**, 394 (1965).*

7 Kubota, Y., Escaño, M. C. S., Nakanishi, H. & Kasai, H. Crystal and Electronic Structure of Li15Si4. *J. Appl. Phys.* ***102**, 053704 (2007).*

8 Ulvestad, A., Mæhlen, J. P. & Kirkengen, M. Silicon Nitride as Anode Material for Li-Ion Batteries: Understanding the SiNx Conversion Reaction. *J. Power Sources* ***399**, 414-421 (2018).*

9 Ogata, K. K., Salager, E., Kerr, C.J., Fraser, Ducati, C., Morris, A.J., Hofmann, S. & Grey, C.P. Revealing Lithium–Silicide Phase Transformations in Nano-Structured Silicon-Based Lithium Ion Batteries *via in Situ* NMR Spectroscopy. *Nat Commun* **5**, 3217 (2014).
